# Supplementary material for: End Sequence Analysis Toolkit (ESAT) expands the extractable information from single-cell RNA-seq data
Source: Genome Res. 2016 Oct;26(10):1397–410. doi: 10.1101/gr.207902.116 (PMC5052061; doi:10.1101/gr.207902.116)
Supplement: Supplemental Material [file supp_gr.207902.116_Supp_Materials.tar.gz › ESAT-esat_v0.1/doc/html/classjaligner_1_1matrix_1_1_matrix_loader_exception.html]

ESAT: jaligner.matrix.MatrixLoaderException Class Reference


ESAT


- Main Page
- Related Pages
- Packages
- Classes
- Files

- Class List
- Class Index
- Class Hierarchy
- Class Members

All Classes Namespaces Files Functions Variables Enumerator Pages

- jaligner
- matrix
- MatrixLoaderException

Public Member Functions |
List of all members

jaligner.matrix.MatrixLoaderException Class Reference

Inheritance diagram for jaligner.matrix.MatrixLoaderException:

[legend]

Collaboration diagram for jaligner.matrix.MatrixLoaderException:

[legend]

|  |  |
| --- | --- |
| Public Member Functions | |
|  | MatrixLoaderException (String message) |
|  | |

## Detailed Description

Author
:   Ahmed Moustafa (ahmed.nosp@m.@use.nosp@m.rs.sf.nosp@m..net)

## Constructor & Destructor Documentation

|  |  |  |  |  |  |
| --- | --- | --- | --- | --- | --- |
| jaligner.matrix.MatrixLoaderException.MatrixLoaderException | ( | String | *message* | ) |  |

Parameters
:   |  |  |
    | --- | --- |
    | message |  |

---

The documentation for this class was generated from the following file:

- java/jaligner/matrix/MatrixLoaderException.java


---

Generated on Fri Aug 15 2014 15:11:06 for ESAT by  

 1.8.7
